# Mobile Autonomous Sensing Unit (MASU): A Framework That Supports Distributed Pervasive Data Sensing

**DOI:** 10.3390/s16071062

**Published:** 2016-07-09

**Authors:** Esunly Medina, David Lopez, Roc Meseguer, Sergio F. Ochoa, Dolors Royo, Rodrigo Santos

**Affiliations:** 1 Department of Computer Architecture, Universitat Politècnica de Catalunya, Barcelona 08034, Spain; esunlyma@ac.upc.edu (E.M.); davidanl@ac.upc.edu (D.L.); dolors@ac.upc.edu (D.R.); 2 Computer Science Department, Universidad de Chile, Beauchef 851, Edificio Norte, 3er Piso, Santiago 8370459, Chile; sochoa@dcc.uchile.cl; 3 Department of Electrical Engineering, Universidad Nacional del Sur—CONICET, Bahia Blanca 8000, Argentina; ierms@uns.edu.ar

**Keywords:** distributed pervasive data sensing, pervasive monitoring, mobile collaboration, software development framework

## Abstract

Pervasive data sensing is a major issue that transverses various research areas and application domains. It allows identifying people’s behaviour and patterns without overwhelming the monitored persons. Although there are many pervasive data sensing applications, they are typically focused on addressing specific problems in a single application domain, making them difficult to generalize or reuse. On the other hand, the platforms for supporting pervasive data sensing impose restrictions to the devices and operational environments that make them unsuitable for monitoring loosely-coupled or fully distributed work. In order to help address this challenge this paper present a framework that supports distributed pervasive data sensing in a generic way. Developers can use this framework to facilitate the implementations of their applications, thus reducing complexity and effort in such an activity. The framework was evaluated using simulations and also through an empirical test, and the obtained results indicate that it is useful to support such a sensing activity in loosely-coupled or fully distributed work scenarios.

## 1. Introduction

Pervasive monitoring allows capturing and characterizing people’s practices and patterns in various scenarios, such as learning, shopping or homecare. This monitoring requires the capture of data from different kinds of sensors, across the different situations and contexts that the people might encounter in their everyday lives. Although smartphones can be used to address this activity, these devices still have some limitations to perform pervasive monitoring; for instance: (1) their sensors are not very accurate; (2) the continuous sensing process involves a high energy cost [1], and their computing power is usually not enough to perform computationally intensive classifications and inference tasks (e.g., speech or image recognition) [2].

These limitations have promoted the appearance of both collaborative sensing techniques that help improve data quality and save energy [3,4,5,6], and also software platforms that help developers create monitoring applications for smartphones. Most these solutions involve the use of centralized components, or assume homogeneity of the devices’ capabilities or stability of the communication links among the participants [7,8,9]. These assumptions do not represent a limitation for conducting some monitoring activities (e.g., crowdsensing [10] or participatory sensing [11]); however, they do for monitoring collaboration scenarios where the smartphone users perform loosely-coupled mobile interactions [12]; for instance, firemen performing first response activities or students involved in mobile learning activities.

In order to support the development of distributed pervasive monitoring applications for this particular niche (i.e., loosely-coupled mobile work), we present a framework named Mobile Autonomous Sensing Unit (MASU) that is able to collect and share data among devices involved in smartphone-based pervasive sensing. Although this framework was initially conceived to support pervasive monitoring in mobile learning contexts, it can also be used to perform pervasive monitoring in a variety of work scenarios.

The MASU framework was evaluated to determine its performance and usage costs in terms of energy, computation and network traffic. The evaluation included simulations and also an empirical test. The preliminary results indicate that this infrastructure is not only useful for data gathering and sharing, but also for reducing the battery consumption of the devices involved in the sensing tasks.

The next section presents and discusses the related work. Section 3 describes the design of the proposed pervasive sensing framework, including its architecture, services, interaction protocols, messages and data retrieval mechanism. Section 4 and Section 5 describe the evaluation process using simulation and an empirical test, respectively, and discuss the obtained results. Section 6 presents a comparison between well-known sensing frameworks and the one presented in this paper, by showing similarities and differences. Finally, Section 7 presents our conclusions and possible future work.

## 2. Related Work

Pervasive applications that monitor loosely-coupled mobile work should consider the dynamism of the interaction context, in which smartphone users can be while performing a collaborative activity. Therefore, pervasive data sensing solutions should provide services for implicit and explicit data sharing, and these services should work in both infrastructure-based and ad hoc networks.

Participatory sensing [2,11] usually involves the voluntary cooperation between smartphone users in order to collect, analyse and share information about their local context. These processes usually require explicit participation of the user, who is actively involved in the data collection process. An example of this voluntary information sharing is when an individual uses his smartphone to take a picture, provide descriptions of his particular context (e.g., in a meeting, cycling, etc.) or tags his current location (e.g., my favourite cafeteria, my brother’s place, etc.). Similarly, mobile crowdsensing [10,13,14] also requires user intervention to provide sensor information. However, it reuses user-entered data from Internet services and social networking sites. These capabilities have allowed people to become active participants of these processes and get a benefit for that. Some services based on these sensing paradigms allow people, for example to identify opportunities for hitchhiking [15] or to evaluate their personal security [16]. Typically, these sensing approaches use infrastructure-based communication that allows mobile sensors to access centralized data repositories, which are in charge of supporting the data sharing process. These approaches are not particularly designed to perform opportunistic sensing, where heterogeneous mobile devices (i.e., devices with distinct sensing capabilities) collaborate to provide each other with contextual data that each device alone could not otherwise sense. For this reason, a different sensing approach is also necessary. Opportunistic sensing [2,6], provides a method to capture contextual information automatically from sensors available in the smartphone. In this case, the user is not directly involved in the data collection process and the information is sensed unobtrusively.

Although there are various applications that provide specific solutions for addressing particular problems, most of them are not easy to use in other contexts, and also require specialized development expertise. For this reason, the development of these types of applications requires to address various challenges related to the limitations of the communication networks and types of interactions in certain contexts, such as unstable communication links, devices heterogeneity, energy constraints, user mobility patterns, etc.

There are also platforms that consider unstable communication scenarios and provide interaction autonomy to the devices participating in a sensing activity, for instance, by using mobile ad hoc networks (MANETs). Nevertheless, they do not take advantage of long-range communication infrastructures when they are available (e.g., Internet connections, cellular networks, etc.) [8,17], which limits the system capability to interact with remote components when they are available. Given the communication in MANETs is usually unstable, there are some proposals (e.g., to support mobile sensing task scheduling [9,18]) that are focused on reducing the data missing rate of mobile nodes, and thereby increasing the gathering rate of the required sensing data.

Some other platforms, like Remora [19] and C-SPINE [20] are designed to exploit device proximity through body sensor networks (BSN). Neighbouring BSNs can opportunistically perform freeriding by using the overheard data from all in-range sensors. The contrary approach is adopted by middleware, like MobIoT [21], which support mobile collaborative sensing at a large scale. However, most these middleware have scalability limitations that are addressed by controlling the participation of redundant sensing devices. These solutions are limited to support pervasive sensing since they compel software designers to choose between short- or large-range data sensing.

Using a hybrid approach platform like METIS [22] (an adaptive smartphone-based sensing system) it is possible to support social sensing by combining smartphones with other devices. This platform decides whether to perform sensing tasks on the local smartphone or on fixed remote sensors, considering the energy costs and the mobility patterns of the user. Following the same line, the middleware proposed in [23] supports collaborative sensing by allowing smartphones to delegate part of their sensing activities to other nearby devices. This leads to an overall reduction in the battery drain of the group of devices involved. Similarly, the CoMon [8] platform supports cooperation, by sharing sensed data among nearby smartphone users, to address the energy drain problem caused by continuous sensing and processing tasks required by monitoring applications. This platform is focused on the detection of potential collaborators (mobile users) and trying to maximize the mutual benefits for the people involved. These last three platforms only try to maximize the benefits of collaborative sensing (in terms of energy consumption) by using smartphones. The lack of capability to involve heterogeneous devices limits their support for pervasive sensing.

Some other platforms, like EasiSee [24], have addressed quite well the sensing scenario by adopting a pervasive approach; in this case, for counting and classifying vehicles in real-time. However, the solution is ad hoc to the problem being addressed (e.g., it considers sensing activities conducted only by a camera and magnetic sensors), which limits its usefulness in other application scenarios.

Provided that pervasive monitoring usually entails diverse types of sensing devices and is dynamic in terms of communication support and users mobility patterns, the monitoring platform should also provide context-awareness [25] and support for device heterogeneity (i.e., allowing interoperability and considering hardware and energy limitations). In this sense, most of the platforms described in this section include centralized components, require a stable communication link to access remote resources, do not support heterogeneous devices or are specific for a certain application domain. Any of these issues jeopardize the suitability of the platforms to support pervasive sensing in various application domains. Next section presents the MASU framework, which is proposed to deal with these issues.

## 3. The MASU Framework

The MASU framework supports opportunistic mobile collaborative sensing activities performed over dynamic and distributed communication scenarios that include both stable and unpredictable communication links. This framework is based on what we call MASU units (potentially mobile nodes), which are smartphones or other devices that run the MASU software infrastructure and interact among them in a peer-to-peer fashion. These nodes not only can work autonomously and perform independent sensing tasks, but also interact opportunistically with other units to perform collaborative sensing. In this sense, the framework allows nodes to act as both consumers and providers of sensing services while preserving their autonomy. The collaboration among a group of MASU units allows the provision of complex and high quality sensing services that could not be provided by individual sensing devices, due to limitations in their capabilities (e.g., CPU, memory, sensor quality, etc.) or to the high cost involved in the sensing tasks. For this reason, the interaction between various units enables a number of collaborative sensing services that are beneficial for the overall group of devices involved in terms of hardware resources, energy consumption and information quality.

In order to model a variety of processes and services involved in a collaborative sensing activity, the MASU framework defines a number of roles played by different units. A role can be seen as a particular set of services provided and/or consumed by a specific unit. A MASU unit can play one or more roles within a collaborative sensing activity. Moreover, it can have various instances of the same type of role activated at the same time. A unit can also participate in a number of parallel collaborative sensing activities, interacting simultaneously with units that are part of different activities and playing the same or different roles in them. In other words, a MASU unit can be seen as an autonomous node playing one or more roles in various work sessions.

**Figure 1:**
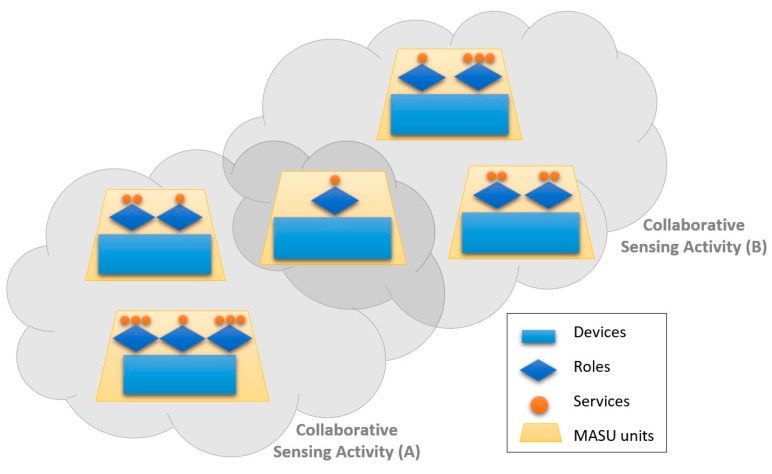
Overview of the MASU work scenario.

Figure 1 shows an overview of the MASU work scenario, when two collaborative sensing activities are being performed among a group of units. It shows two different groups of units that, according to the roles played by them, provide and/or consume diverse services within the activity they are participating. In addition, one of the units is participating in both sensing activities so that it can contribute to and benefit from both of them.

The process to conceive, develop and evaluate the MASU framework was inspired in the design science research approach [26], which is a problem solving process that involves seven steps (guidelines) for finding a solution to the stated problem (an information system). Following these guidelines we defined the MASU design (guideline 1) for addressing the pervasive sensing problem (guideline 2). We evaluated the performance and capability of such a design using simulations and also through an empirical test using a MASU-based application (guideline 3). The evaluation results indicated the framework was able to manage the device heterogeneity in a distributed way, considering various types of communication links among nodes; which represents a clear contribution for the development of pervasive sensing solutions (guideline 4). The MASU design was represented using contextualized component diagrams (guideline 5), and these design solutions were revised through periodic formal technical reviews [27] that helped us find effective solutions to the design challenges of the system (guideline 6). Finally, the design results were communicated to the technical audience involved in the MASU evaluation process in order to help them make an effective use of the framework (guideline 7).

The design evaluation methods used during the development of MASU were diverse; each one was chosen depending on the development stage of the product. Particularly, architecture analysis (design inspections [27]) was used during system conception and evolution. Then simulations were utilized to determine the performance and capability of the system before implementing it. These properties of the system were verified using black-box testing, and its usefulness was evaluated using a real application scenario; particularly, a MASU-based application was developed to conduct a pervasive sensing process. The steps of the design science research approach followed in the development and evaluation of the MASU framework are described more in details in the next subsections.

### 3.1. System Architecture

**Figure 2:**
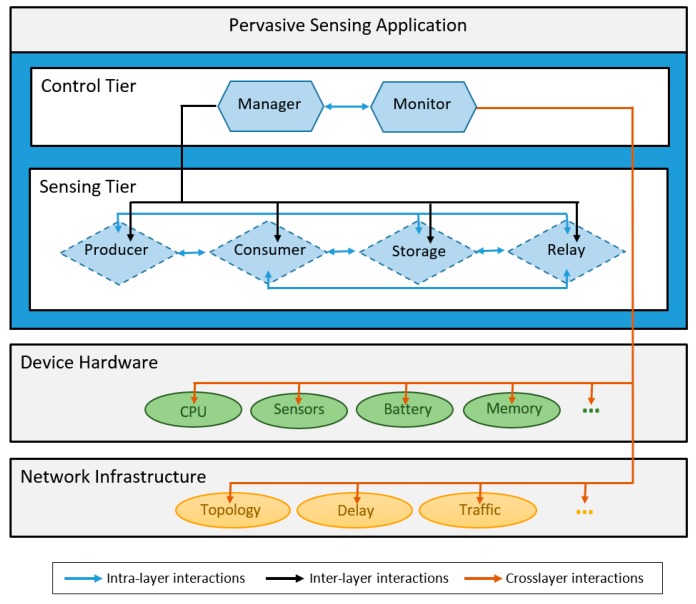
Architecture of the MASU Framework.

The architecture of the MASU framework includes two separate layers (Figure 2): the *Control Tier* and the *Sensing Tier*, which interact among themselves and define two different categories of roles. This is a crosslayer architecture that enables interactions with other layers of the computing system, and benefits from the information that other layers may provide (i.e., hardware and network information). The particular pervasive sensing applications developed over MASU are in the upper layer, and therefore they consume the services provided by the framework.

The *Control Tier* provides all the services required for the management and monitoring of the overall collaborative sensing activity, coordinating the tasks within the activity and also among different activities, if required. This layer is also in charge of managing the use of resources within each sensing device (MASU unit).

This layer includes two roles: Manager and Monitor, which interact among themselves to coordinate the operation of this tier and provide the collaborative sensing services. Therefore, the Control Tier performs role selection and activation functions, and it also has control over the Sensing Tier. This layer also monitors the state of the device’s hardware components, such as battery level, processor load, memory available and quality of the sensors available (hardware monitoring). Moreover, it keeps track of events related to the underlying network infrastructure, including topological changes, network traffic, congestion and delay (network monitoring). Such monitoring allows the MASU units to be context-aware and make appropriate role selection and activation decisions, as well as to adapt to the unpredictability of dynamic environments, activating or deactivating roles accordingly.

The *Sensing Tier*, which is in charge of performing the specific data sensing and sharing tasks, has four distinct roles: Producer, Consumer, Storage and Relay. These roles interact among themselves enabling the provision of complex data sensing services.

### 3.2. Node Structure

**Figure 3:**
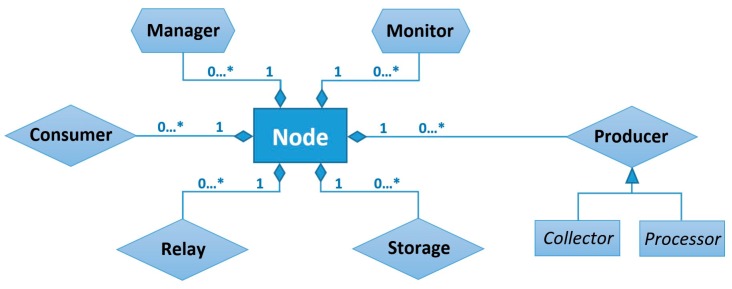
Structure of a MASU node.

The node running the MASU platform can play one or more of the roles defined in the framework. Figure 3 represents the role composition structure of a MASU node.

A node can also have various instances of the same role activated at the same time, but in different sensing sessions. These roles are dynamic and can evolve over time according to the characteristics of the network infrastructure, the mobility of the nodes, or changes in the number of nodes involved in the activity.

In order to conduct a collaborative sensing activity, it is necessary that at least two nodes are willing to collaborate, and such collaboration implies some benefit for the participating nodes. Otherwise, the MASU units would be working independently, in autonomous mode. A node performing sensing activities independently in a stand-alone fashion, would be at the same time Producer and Consumer of its own sensing services. It would, therefore, only require the activation of particular Producer roles for the required services and the corresponding Consumers of such services. By contrast, in a scenario where a set of nodes is working collaboratively to perform a sensing activity, there can be diverse combinations on the number and type of roles that must be activated in different nodes, depending on the requirements of the activity and on the characteristics and capabilities of the participating devices. However, this collaborative sensing activity would require that at least one Manager, one Monitor, one Producer and one Consumer roles be activated in the activity.

Typically, most nodes collaborating in a sensing activity will have at least a Consumer role activated, because we assume that they will be interested in at least part of the sensed data. Nevertheless, if some of the MASU units participating in the activity are Internet of Things (IoT)-based devices, there can be cases where they would require the activation of a Consumer role (e.g., a shared display receiving an image), but in other cases, they would only provide sensing services and would not have any Consumer role active (e.g., sensors in smart buildings).

There can be two different kinds of Producer roles: Collector and Processor, depending on the type of information that they generate or in the method used by them to obtain it. Moreover, Relay and Storage roles can be activated depending on the network conditions and on the characteristics of the sensing activity and of the participating nodes.

### 3.3. Roles and Services

MASU units playing some roles can interact with each other by subscribing to the services provided by specific roles. When a unit subscribes to a particular role, it is then, subscribing to all the services provided by such a role. The role *manager* acts as coordinator of the collaborative sensing activity, and there can be various managers coordinating the activity. The *monitors* supervise the performance of MASU units. The *producers* perform sensing tasks using any of the sensors available in the device. The *consumers* are typical service consumers that use the data sensed by the producers. They can access this data directly from the producers, and also through storages or relays. The *storage* acts as an active data repository that can be subscribed to the data obtained from the producers. The *relays* are in charge to retransmit recent data generated by producers, to consumers that lost or could not receive the data sent by the producers properly.

**Figure 4:**
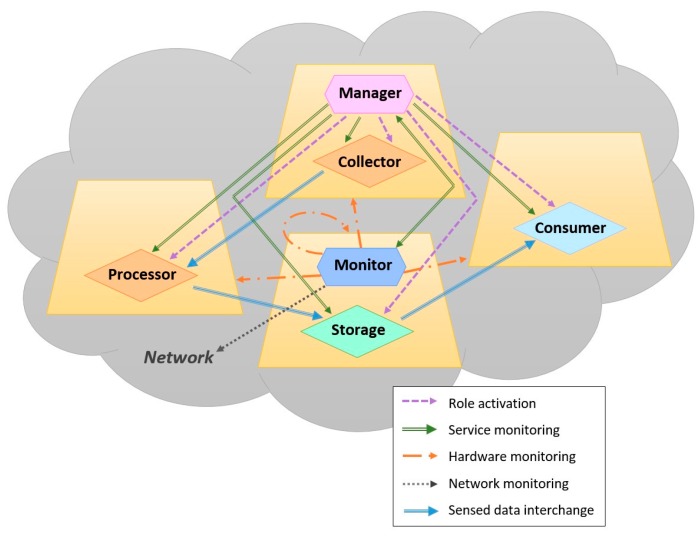
Interactions between MASU roles.

Figure 4 shows an example of a collaborative sensing activity involving four MASU units and various roles. The arcs represent the interactions between roles. According to Figure 4, the Manager activates all the roles present in the activity and also performs service monitoring over them. On the other hand, the Monitor performs network monitoring, service monitoring over the Manager and hardware monitoring over the four participating units. In the activity we have two Producers: one acting as Collector and the other as Processor. In this case, the Processor receives the data sensed previously by the Collector, performs some classification or inferences tasks and sends the resulting information to the Storage. Finally, the Storage sends this information to the Consumer.

### 3.4. Internal Mechanisms

Three internal mechanisms govern the dynamic of the individual and collective work of the mobile units. Next we briefly explain these mechanism.

#### 3.4.1. Mechanisms for Role Selection and Activation

Once the set of devices that will take part in the collaborative sensing activity is determined, all the participating units share their hardware capabilities. Then, they have to select and activate the local roles in each tier. In this sense, we distinguish between two basic operations: (i) Role Selection and Activation in the Control Tier and (ii) Role Selection and Activation in the Sensing Tier. MASU supports dynamic centralized or distributed approaches for conducting this operation. Table 1 shows a summary of the methods followed for the selection of roles in the different Tiers, according to the role chosen for the Control Tier. If the role activated in the Control Tier is Dynamic Centralized, the selection of roles in this Tier will be determined by the results of the election algorithm. Furthermore, due to the fact that in this architecture we only have one manager, the selection of roles in the Sensing Tier will be determined by such a manager. Nevertheless, if the role architecture of the Control Tier is distributed, all the units that are part of the collaborative sensing activity and that are active and have connectivity will play the roles of the Control Tier. In addition, the Selection and Activation of roles in the Sensing Tier will be determined by the results of the consensus algorithm.

#### 3.4.2. Cost Function and Resource Optimization

Before selecting and activating roles in the Sensing Tier, the Manager uses the information received from the MASU units to run a Cost Function. The output of this function will determine whether it would be beneficial for the whole group of units to work collaboratively or not. According to such output, the Manager will activate or not the Role Selection and Activation processes in the Sensing Tier. Consequently, if the output of the Cost Function is negative, there will not be collaborative activity and all the units will work independently. The Cost Function determines the viability and usefulness of performing collaborative sensing.

The Cost Function is also associated with the results of the Resource Optimization Algorithm (ROA), which helps optimize the use of the resources of the whole group of participating units. The ROA algorithm runs every time that the MASU framework wants to initiate the Role Selection and Activation process in the Sensing Tier. Such an algorithm influences how roles are selected, calculating the estimated costs of the services provided by each one of the roles, and considering the specific requirements of the activity and the characteristics and state of the devices. The ROA determines, for a group of units, who has to activate a given role, who has to collect data from specific sensors and share the results with the rest of the members, etc. The ROA will also decide, between these two Producer units, which one have to capture data from microphone and which one have to sense GPS data. As a result, the rest of the units acting as consumers will deactivate their sensors and wait until they receive the sensed information from these two producer units.

#### 3.4.3. Fault Tolerance Mechanisms

Due to the dynamism and uncertainty of some network infrastructures, the MASU defines various fault tolerance mechanisms to deal with changes and failures in active roles that are taking part in a collaborative sensing activity. Such mechanisms can be classified into two categories: tolerant to role failure and tolerant to resource limitations. In order to detect a role failure, the framework monitors the state of each one of the roles that were activated to perform the activity. As explained previously, the manager role monitors the state and behaviour of the services offered by all the other roles. Similarly, both manager and monitor perform mutual service monitoring on each other.

Concerning the mechanisms of tolerance to resource limitation (e.g., battery, memory or CPU) the monitor and manager keep track of any event in the collaborative activity: new units join, some disappear, others have poor network connectivity or run out battery, CPU or storage capacity, etc. If any of these changes occur, the Cost Function must be executed to determine the viability and usefulness of the collaborative sensing activity. If the output of this function is positive, the MASU framework will restart the Role Selection and Activation mechanisms for the Sensing Tier, reassigning roles as appropriated according to the results of the ROA algorithm.

In the case that many new units join the collaborative sensing activity, it is possible that the cost required to share the sensed data is too high, and therefore the output of the Cost Function is negative. This situation would imply that the collaborative sensing activity is not beneficial for the overall group of participating units so that it has to end. However, this situation could be solved by creating two parallel sensing activities.

### 3.5. Control Messages

In order to support the interaction among units, the MASU framework defines the following six types of messages of the Control Tier. The *Unit Detection* messages that are used to detect the units that are present in the collaborative sensing activity. Therefore, these messages are used to monitor changes in the composition of the activity, regarding the units and roles that are present in such activity. The *Device Information* messages that are used by the units to share information about their hardware capabilities, such as battery level, processor load or available memory. The *Election* messages that are sent by the distributed leader election algorithm, used to select the manager and monitor roles in a dynamic centralized role architecture of the Control Tier. The *Consensus* messages that are used in a distributed role architecture of the Control Tier, so that all the managers could agree on the selection of roles in the Sensing Tier. The *Role Activation* messages are only used in case of a Dynamic Centralized role architecture of the Control Tier. They are sent by the manager to activate roles in the Sensing Tier or to activate the Monitor if it fails. Finally, the *Network Monitoring* messages represents a special type of message that is necessary for the network monitoring functions performed by the monitor.

### 3.6. Data Retrieval Mechanism

The interactions between the different roles of the Sensing Tier enables the MASU units, participating in the collaborative sensing activity, to collect and share data so that all the units can have the information required by the activity. These interactions are mediated by the Collaborative Sensing Module (CoSM), which is in charge of the data retrieval and allows the differential activation of each one of the roles of the Sensing Tier (i.e., consumer, producer, storage and relay).

**Figure 5:**
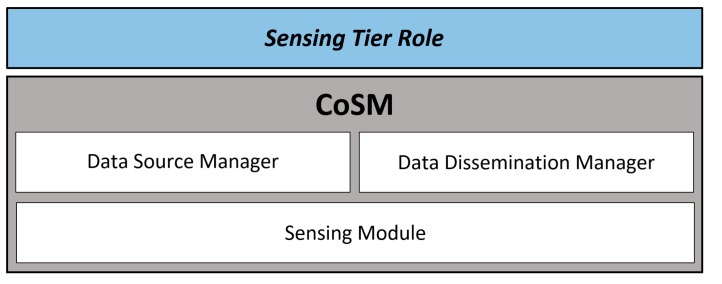
Structure of the Collaborative Sensing Module (CoSM).

This component offers four basic services for service discovery [28] and information distribution: Publish, Find, Subscribe, and Data Dissemination. Figure 5 shows the structure of CoSM, which is composed by three main modules: the Sensing module, the Data Source Manager and the Data Dissemination Manager. They are responsible of the services that enable the differential activation of the roles in the Sensing Tier. The Sensing module interacts with the other two to determine the type of data that a particular unit has to sense and the method that it will use to obtain such data. Next we explain more in detail these components.

#### 3.6.1. Sensing Module

The Sensing Module (SM) supports the data collection process. The data can be collected from the sensors available on the MASU unit as well as retrieved from other units. For the MASU framework a sensor is any hardware or software component that act as source of information. Consequently, the SM module calls a number of services for accessing any type of sensor available on the unit.

This module can obtain raw data, e.g., from the unit’s physical sensors, but also high level information from other types of sensors. In the former case, a Collector role will be activated, while in the latter the role played by the unit will be a Processor. The SM also enables the use of data shared by other units as data source and establishes (for every sensor in the unit) the way how it will obtain the corresponding data. This way, the activation of particular Producer or Consumers roles can take place. Accordingly, the SM defines two basic data collection methods: direct and indirect. In the direct method the unit is responsible for capturing data without relying on any other source. In the second case, the sensor receives and processes data that has been previously collected by other units. In the former case the unit will play a producer role, whereas in the latter it will be a Consumer.

The SM allows both, remote and local activation of the all the sensing services. This fact facilitates the activation of roles in the MASU units by the manager for collaborative sensing activities as well as the independent operation of the units for individual sensing tasks. The SM also allows flexible configuration of the sensing frequency and waiting times.

The data collection methods specified by the SM allow a selective distribution of data from different sensors. That is, the MASU framework facilitates a flexible selection of the collection methods that will be used for each one of the sensors available on the units. For example, it can decide to use a direct method to capture data from the accelerometer of the unit but to use an indirect method to capture GPS data.

**Figure 6:**
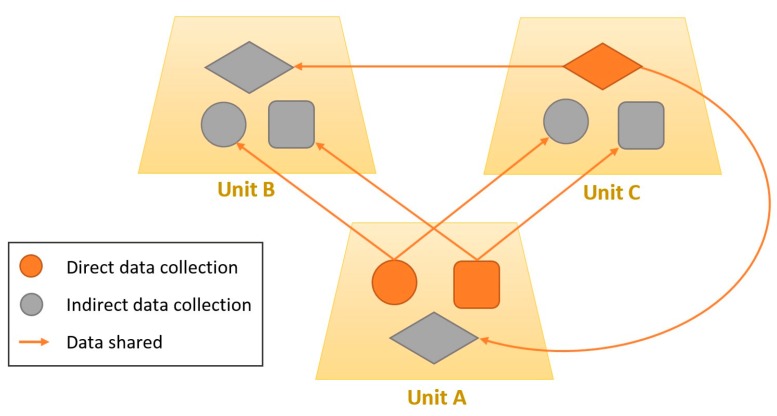
Example of data sharing between units.

Figure 6 illustrates a data sharing process conducted by three different units. These units have activated three kinds of sensors. Unit A shares data from two sensors. This data was collected directly by the unit, which means that these sensors were activated in direct mode. On the other hand, this unit also has a sensor activated in indirect mode, receiving data from one of the sensors of Unit C. In addition, Unit B has all its sensors activated in indirect mode. Then, this unit will receive all the data from Units A and C.

#### 3.6.2. Data Source Manager

The Data Source Manager (DSM) is in charge of specifying the sensors that the unit will use to collect the input data. The DSM enables the units to capture diverse types of data from different sensors so that the MASU units can sense different kinds of information and share it with others.

The MASU framework supports diverse sensors or data sources, such as IoT devices, information repositories, sensors in smart buildings and sensors embedded in commercial smartphones. These sources must be able to provide information that is relevant for the applications and users (in our case, information that is relevant for pervasive monitoring and awareness). Moreover, as stated in [25], any modern mobile ubiquitous system must provide context-awareness and therefore information about the environment that is providing services to the users (in our case, the MASU unit). For this reason, the proposed framework supports a wide range of data sources that are necessary to provide context-awareness about the MASU units (what we previously called hardware monitoring) as well as useful information for pervasive monitoring in learning scenarios. Next, we specify the different categories of data sources that are supported:

- *Physical sensors*: We can differentiate between three kinds of physical sensors: hardware sensors (e.g., accelerometer, GPS or compass), communication sensors (correspond to built-in communication interfaces like Bluetooth, infrared or Wi-Fi), and performance sensors (that determine battery level, or CPU/memory utilization).
- *Virtual sensors*: In this group we consider information that can be obtained from the device’s applications and services; e.g., the screen status, the user’s touch inputs, applications status, log files and notifications.
- *Human-based sensors*: We include in this category any custom application used to collect information that require explicit user intervention. These types of sensors require the participation of a human user to provide information and create new knowledge [29]. Human-based sensors complement the implicit sensing process performed automatically by unobtrusive sensors. This way we can obtain both objective (e.g., a picture, a quantitative datum, etc.) and subjective (e.g., perception, opinion, etc.) information from the user.
- *Context sensors*: These are modules that collect information related to the user context [25] from existing repositories; for instance, the user’s profile, preferences, schedule or performance indicators.
- *Logical sensors*: These sensors provide high-level information and they can combine data from a number of sources. Information from this category usually involves some type of aggregation and processing to interpret the sensed data and contextual information. An example of logical sensors could be a service that interprets raw data from an accelerometer to infer the type of physical activity that the user is performing (e.g., sitting, walking, running, etc.).

In the MASU framework there can be units that act as producers and consumers of the different types of data that these categories of sensors provide. MASU units that use an indirect method to collect input data from any of these sensors will act as Consumers, whereas units that use a direct method will fulfil a particular Producer role. In this later case, the MASU units that sense data using physical, virtual, human-based or context sensors can have a collector or processor role, depending on whether this data require some level of interpretation (e.g., classification, aggregation, processing, etc.) or not. On the other hand, those units that retrieve data from logical sensors to perform further interpretation tasks always play a Processor role.

#### 3.6.3. Data Dissemination Manager

**Figure 7:**
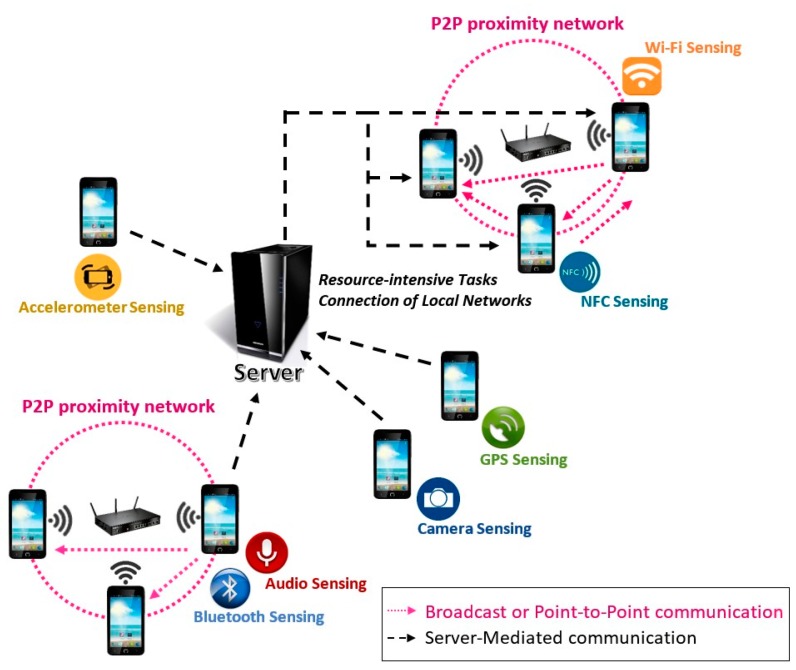
Example scenario of data dissemination.

As shown in Figure 7, the Data Dissemination Manager (DDM) establishes three data dissemination mechanisms: Broadcast, Point-to-Point and Server-Mediated.

The broadcast and point-to-point mechanisms offer different methods to create peer-to-peer proximity networks amongst devices. It allows the MASU units to share information directly among them, without depending on any centralized server. This fact opens up the possibility to integrate the MASU framework with IoT, allowing that the units could interact with nearby networked objects and sensors. In the case of broadcast data dissemination, the data sent by a unit can be received by all the others. On the other hand, the point-to-point mechanism only allow data transfer between pairs of units and therefore only the particular unit that was specified as destination of the data can receive and use it.

Finally, the Server-Mediated data dissemination allows communication between units that are not in the same local network. This mechanism enables data transfer between two independent groups of units connected to different local networks but reachable through the Internet. For instance, this two groups could be units that are participating in two different collaborative sensing activities. Furthermore, the MASU framework can decide to make use of this server to perform some resource intensive aggregation, processing or classification tasks to optimize the use of local hardware resources of the units.

## 4. MASU Evaluation Using Simulations

The first step in the MASU evaluation process was to determine its usefulness and performance under diverse networking, hardware and mobility conditions, by using simulations. Next we explain this process and the obtained results.

### 4.1. Simulations Setup

In this process we used the ns-3 simulator [30], since it allows us to represent diverse scenarios and collect a number of metrics with the purpose of assessing the impact of the underlying network infrastructure, as well as the mobility patterns of the MASU units, on the framework performance. The ns-3 enables the configuration of the nodes that run the MASU framework, the communication network that supports them and the physical space where they are placed. Consequently, the hardware capabilities of the nodes, their wireless network interfaces, physical position and mobility patterns were configured using this simulation tool. We performed 20 simulations for each particular scenario, and each simulations lasted 20 min.

#### 4.1.1. Nodes and Physical Space

We defined a 360 m × 360 m outdoor area to place the nodes. The size was set not only to allow the mobility of the nodes and the creation and evolution of different network topologies, but also considering that diverse everyday activities can take place in such a space.

In this area, we placed 40 mobile nodes that represent the autonomous units running the MASU framework. We initially considered all nodes similar, having the same technical features. Therefore, these simulations do not consider the effect of device heterogeneity, which will be addressed in the evaluation of the system prototype. Particularly, we configured the nodes to have the capabilities of a phone equivalent to an iPhone 5 (Apple Inc., Cupertino, CA, USA) or a Samsung Galaxy S5 (Samsung Electronics Co., Suwon, Korea). These devices have an effective Wi-Fi communication range of approximately 80 m in open areas. Table 2 summarizes the general parameters configured in the ns-3 for the simulations.

#### 4.1.2. Mobility Patterns

The nodes’ movements were modelled using the BonnMotion tool [31], which includes well-known models that represent people’s mobility patterns [32]. For the simulations, the nodes’ speed was set between 0 m/s (static nodes) and 1.5 m/s (walking speed) and three mobility patterns were used: RandomWalk, SLAW, and Nomadic. Table 3 shows the parameters used for the setup of the mobility models considered.

This configuration helped us simulate a dynamic network, where some existing communication links can be lost and new links can appear. Such a configuration represents a realistic scenario (e.g., opportunistic collaboration in a university campus) where people can move within the physical space, and eventually interact with other people that they meet.

#### 4.1.3. Network Infrastructures

We configured the simulator to use three different types of networks to support the communication between the nodes: Access Point (AP)-based, Terminal-to-terminal (T2T) and MANET. These wireless network infrastructures, based on the IEEE 802.11 standards, were set according to the proposals presented in [33,34], and they were adapted to be simulated on ns-3. Table 4 shows a detail of the configuration of the ns-3 simulator for the different types of User Datagram Protocol (UDP) messages interchanged in the three network infrastructures considered.

#### 4.1.4. Role Selection and Activation Methods

The simulations consider Dynamic Centralized and Distributed role architectures. Therefore, we implemented the three methods defined in the framework for Role Selections and Activation in the Sensing and Control Tiers: (i) the manager decides; (ii) the leader election algorithm decides or (iii) the consensus algorithm decides. We implemented a distributed leader election algorithm based on the proposals presented in [35,36]. Similarly, the distributed consensus algorithm used in the simulator was based on [37,38].

### 4.2. Simulation Results

Next we present the simulations results that show the behaviour of the Control Tier of the MASU framework for the different network infrastructures and mobility patterns. It also considers the costs of the actions performed by such a tier, in terms of network utilisation. In order to verify that the Control Tier works as expected, we performed various simulations of the Dynamic Centralized and the Distributed role architecture approaches considered by the MASU framework and compared them with a Fixed Centralized method (used as baseline), where there is no dynamic selection and activation of roles in the Control Tier. For both approaches we implemented real algorithms in order to confirm that the MASU framework behaves as expected. In case of the Dynamic Centralized approach, we implemented a distributed election algorithm for the selection of a Manager every time it fails. Similarly, in the Distributed approach we implemented a distributed consensus algorithm for the selection of a Producer when necessary. Next we present the evaluation of the role architectures of the Control Tier considering different (i) network infrastructures and (ii) mobility patterns.

#### 4.2.1. Considering Different Network Infrastructures

**Figure 8:**
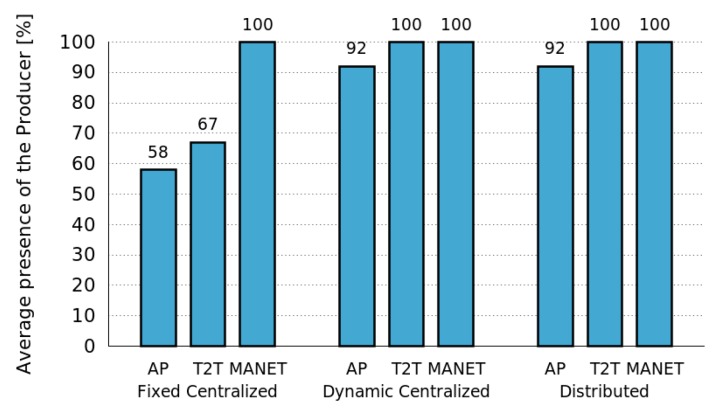
Performance of the Control Tier for different network infrastructures.

For this first set of simulations we used the RandomWalk mobility pattern and evaluated the performance of the MASU framework for various network infrastructures: AP, T2T and MANET. As shown in Figure 8, for AP and T2T networks in the Dynamic Centralized and the Distributed approaches, the average presence of the Producer increases, when no Control Tier was active. Nevertheless, in the Fixed Centralized method the results for both types of networks are not good enough when there is less than 60 and 70 of average Producer presence, respectively. Notice that the results obtained for MANETs are always the same, independently of the approach followed.

These results show that the Dynamic Centralized and Distributed approaches considered in the MASU framework allow one to achieve significant improvements in comparison to a Fixed Centralized approach. Next we evaluate the cost required for such improvements, in terms of the number of messages sent by the algorithms used for dynamic role Selection and Activation.

For this evaluation, we considered the four (i.e., Unit Detection, Device Information, Election and Consensus messages) of the six types of control messages defined by the MASU framework as well as the particular messages required by the routing protocol in MANET networks (routing messages). However, in the Device Information messages, we did not consider the messages required for hardware monitoring because we cannot measure changes of the hardware components of the devices in the simulator. We only considered the Device Information messages sent after a role Selection and Activation process in the Sensing and/or in the Control Tiers takes place. Thus, we only included the messages sent by new network nodes that are unknown for the new Manager selected. Therefore, the number of Device Information messages is associated with the number of nodes that are concurrently active when a new Manager is selected.

**Figure 9:**
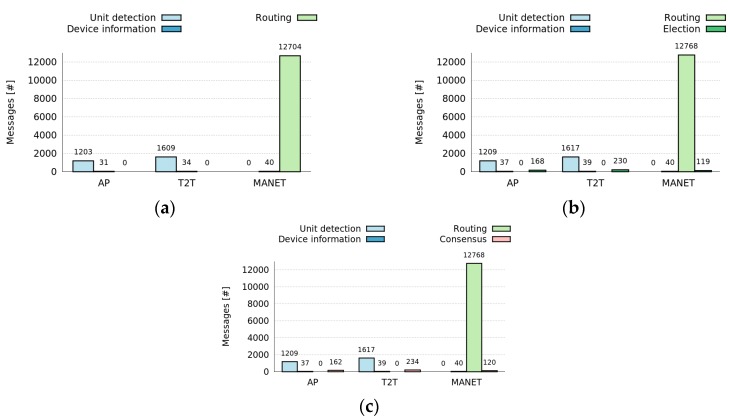
Cost of the different role architectures of the Control Tier for different network architectures. (**a**) Fixed Centralized; (**b**) Dynamic Centralized; (**c**) Distributed.

Figure 9 shows a comparison of the number of messages required for the Dynamic Selection and Activation of roles in Fixed Centralized, Dynamic Centralized and Distributed role architectures of the Control Tier. These figures show that the number of messages is very similar for both Dynamic Centralized and Distributed approaches. Nonetheless, the number of messages is slightly smaller for the Fixed Centralized approach. This can be explained by the fact that in the Fixed Centralized approach, the first node that join the network plays both the Manager and the Monitor roles and when such a node fails, the system stop counting the messages that this node was monitoring.

From Figure 9 we can conclude that the number of messages required by the framework depends on the network infrastructure, and it is positively correlated with the number of nodes of the network. Thus, AP infrastructures produce a smaller number of messages, followed by T2T and MANET networks.

#### 4.2.2. Considering Different Mobility Patterns

**Figure 10:**
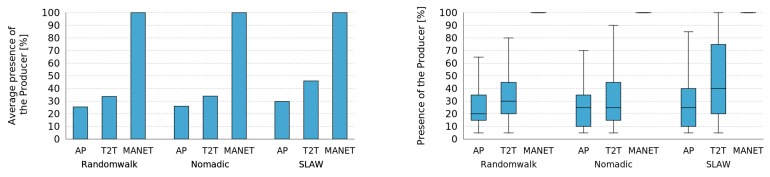
Presence of the Producer for different mobility patterns when no Control Tier is available.

In order to evaluate the impact of the units mobility patterns on framework performance we simulated nodes when there is no Control Tier available. Figure 10 shows the average presence of Producer considering the different network infrastructures and mobility patterns. RandomWalk and Nomadic mobility patterns achieve a similar percentage of presence of the Producer in AP and T2T networks, whereas Self-similar Least Action Walk (SLAW) achieves a slightly higher percentage. Nevertheless, the improvement achieved by the SLAW mobility pattern is not significant.

On the other hand, MANET networks achieve the maximum percentage, regardless of the mobility pattern. These results suggest that more realistic mobility patterns yield to higher presence of the Producer. This can be explained due to the fact that such patterns consider the social tendency of human interactions and therefore people’s inclination to form groups.

**Figure 11:**
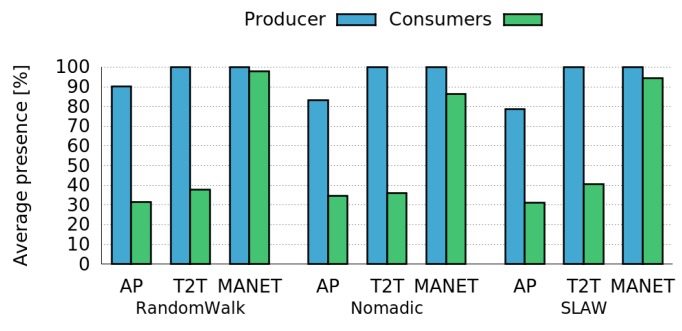
Presence of the Producer and the Consumers for different mobility patterns using an ideal Control Tier.

Then, we evaluated the performance of an ideal Control Tier as well as the average presence of Consumers for the different mobility patterns considered. Figure 11 shows that the presence of Consumers varies slightly across mobility patterns and network infrastructures. In addition, the performance of an ideal Control Tier in terms of presence of the Producer is similar for all mobility patterns in T2T and MANETs, while RandomWalk obtains slightly better results for AP networks. Thus, the variation in the results obtained for different mobility patterns are not significant.

#### 4.2.3. Discussion of the Simulation Results

All previous results suggest that MANET networks can provide an interesting alternative to support pervasive data sensing in dynamic scenarios, since they guarantee higher node presence values (especially, Consumers since the Control Tier of the framework already deals with the presence of Producers), regardless of the mobility patterns considered. This fact means that higher data availability is possible using this types of networks since Consumers can receive data from Producers regardless of the fluctuations in the communication links and the mobility conditions of the nodes.

**Figure 12:**
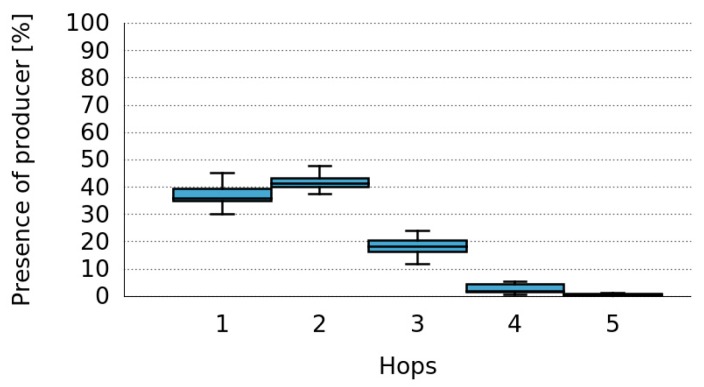
Percentage of presence of the Producer versus the number of hops in MANET networks.

The explanation for this high data accessibility can be found in Figure 12. It shows that more than 50% of the time that the Producer is present in the network it is located more than one hop away from the Consumers. There are even cases where the Consumers receive data from the Producer when it is at four or five hops away.

**Figure 13:**
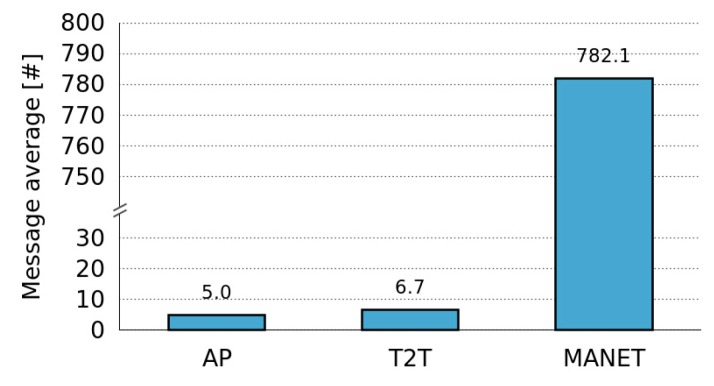
Average number of messages for different network infrastructures.

These benefits of MANETs come at the expense of a higher number of messages that have to be transmitted over the network, as represented in Figure 13. This figure shows the average number of messages that are transmitted over the network for different network infrastructures. The number of messages is significantly higher in MANETs due to the fact that the data messages transmitted from the Producer to the Consumers are UDP unicast (1 to 1). For this reason, the Producer has to send one message to each one of the Consumers that are within the network. Thus, the number of data messages has a direct correlation with the number of active Consumers. By contrast, in AP and T2T networks the data messages are UDP broadcast (1 to all) so the number of messages has a positive correlation with the number of active Producers.

The higher cost related to the number of messages in MANETs is compensated by the fact that these types of networks allow that a higher number of units could receive the information sensed by Producers. However, if the number of Consumers is too high, this cost could lead to network congestion, which would also suppose that the MASU framework will not be able to work properly under those conditions. As a result, it would be necessary to find a solution to deal with scalability problems related with the high number of messages sent through the network in MANET.

## 5. MASU Empirical Evaluation

In order to evaluate empirically the proposed framework, a fully distributed mobile application was implemented using MASU. The framework provides a number of services for automatic collection and collaborative distribution of the data gathered from the device sensors. Such services facilitate the access to the sensors available on the device, enabling data collection and sharing between devices according to the specifications of the Collaborative Sensing Mechanism (CoSM) of the framework.

### 5.1. Role Selection and Activation Methods

The implementation of the Role Selection and Activation processes was done according to the following procedure:

- The Manager is fixed for the duration of the collaborative sensing activity and it is responsible of selecting and activating roles in the Sensing Tier (i.e., Consumer, Producer and Storage), sending messages to other units accordingly.
- When a new node accesses the network, it announces its state, capabilities and the sensing services that can provide. As a result, the Manager knows which sensors are available in the network and therefore which units are suitable candidates to play Producer roles.
- In case that various units meet the requirements to play a particular Producer role, the Manager takes the decision considering the quality of the sensor and the battery level of the device. Thus, the units that have sensors with the highest quality and the highest battery level, will be selected as Producers.
- After a specific Producer role is selected and activated, the Manager automatically activates Consumers of the information sensed by that Producer in all the units connected to the network.
- Consumers can receive information from Producers (i) periodically or (ii) only when the information changes, receiving a notification as well as the new data when it is available.
- Every time a new node joins the network, the Manager starts the Role Selection and Activation process in the Sensing Tier, activating a Consumer role in the new node.
- The Manager selects and activates a unit to act as Storage of the data collected by a specific Producer. This unit stores all the information sent by such a Producer in a SQLite database.
- Consumers receive data from the Storage periodically, according to the data rate specified by the Consumers in the data request.

### 5.2. Data Gathering Methods

Our prototype of the MASU framework implements three different methods for the provision of the data retrieval services (Publish, Find, Subscribe, and Data Dissemination services) included in the CoSM mechanism embedded in the MASU framework. The three methods implemented are: AllJoyn [39], CoAP [40] and GCM [41,42]. Alljoyn and CoAP offer different mechanisms to create a Peer-to-Peer proximity networks, enabling devices to share information directly among them, without depending on any centralized server. By contrast, if the devices are not physically close but they have Internet connectivity, they can share information using the GCM service, which is connected to the CoSM Server. Next we describe the implemented prototypes.

### 5.3. Prototypes Implementation

Two different prototypes were developed in order to evaluate the performance of the framework using various implementation types, network infrastructures and data retrieval mechanisms. Table 5 summarizes the main features of both prototypes. The Prototype I was implemented using the Unity development platform [43], whereas the Prototype II was implemented as a native Android application. Provided that Unity is a cross-layer platform, the Prototype II was deployed for both Android and Windows OS.

Concerning the network infrastructures, the two prototypes provide distinct methods to create peer-to-peer proximity networks among MASU units. The Prototype I allows a group of units to connect to an existing AP, while the other configures automatically the first unit that joins the activity as AP to provide wireless access for the whole group of units. As a result, AP-based (AP) and Terminal-to-Terminal (T2T) networks are established, respectively. In both prototypes, the first unit that joins the network uses AllJoyn to create the peer-to-peer proximity network. Such a unit will also act as Manager for the overall duration of the collaborative sensing activity. Therefore, we have a Fixed Centralized role architecture of the Control Tier without any mechanism for the dynamic selection and activation of roles in such a tier. For this reason, these prototype implementations were useful only to evaluate the Sensing Tier of the MASU framework.

### 5.4. Evaluation Results

The performance of the Sensing Tier, for both prototypes, was evaluated in terms of resource consumption and considering heterogeneous devices. In contrast to the simulation tests, the prototype evaluation considered devices with diverse capabilities. In order to do that, we deployed the prototype in different Android devices. We used smartphones and tablets with different OS versions and various kinds of CPU, battery and sensor chips. The features of the different types of devices used are detailed in Table 6. This device heterogeneity allows us to gain some insights about the changes in the performance of the framework across devices.

The evaluation considered number of tests where some of devices sensed the environment and shared the obtained data, and others only received the sensed information. The duration of all the tests was 20 min, and in every test the battery of all the devices had the same charge level (100%). The tests performed consider an outdoor space, where the devices are moving arbitrarily at walking speed.

In the evaluation of the Prototype I, the devices remained within the coverage zone of a fixed AP. By contrast, in the evaluation of the Prototype II, the devices are never outside the coverage zone of the device that acts as AP or their mutual coverage zone. As a result, both types of tests do not consider dynamic network topologies or diverse mobility patterns.

#### 5.4.1. Benefits of Collaborative Sensing

Next, we present various tests done using the Prototype I to assess the usefulness of sharing data among MASU units, instead of working autonomously. Although this assessment only considers the advantages of collaborative sensing in terms of the battery consumption, other aspects must also to be taken into account, such as better information quality and social welfare (the benefit of the entire community of users).

**Figure 14:**
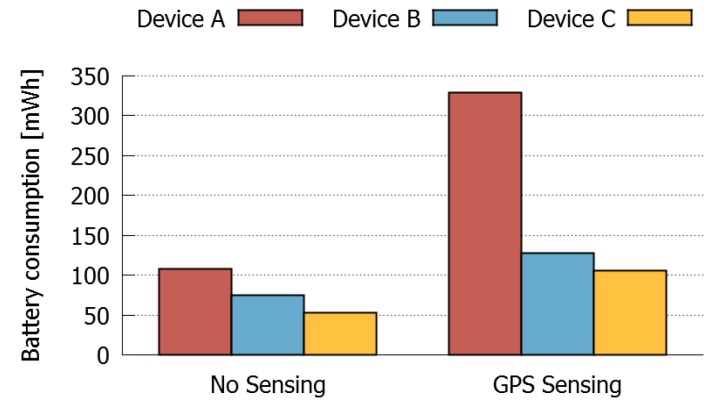
Sensing costs for different devices.

First, we evaluated the battery consumption produced by a sensing activity in different devices. Figure 14 shows the results obtained when the devices are not performing any sensing or sharing activities, and also when they are sensing GPS data. This graph indicates that there is a significant cost in battery lifetime, due to the sensing operations. Moreover, this cost differs across devices, having the device A the highest cost. This difference in battery consumption can be caused by differences in the versions of the operating system or the GPS sensing chips.

**Figure 15:**
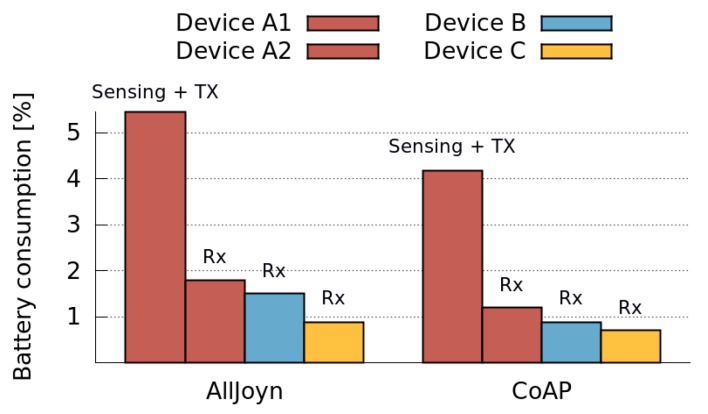
Comparison of the overall collaborative sensing costs of AllJoyn and CoAP for different devices.

Then, we evaluated the costs involved in a collaborative sensing activity considering heterogeneous devices. Therefore, we compared the total battery consumption when sensing data and transmitting it using the two local data retrieval mechanisms: AllJoyn and CoAP. We conducted two different sets of experiments, where four different devices share GPS data, using AllJoyn and CoAP, respectively. Figure 15 depicts the results when the device A1 is sensing and transmitting GPS data (Producer role) and devices A2, B and C are receiving it (Consumers). As expected, the energy consumption is higher in the transmitting device than in the receiving ones for both, AllJoyn and CoAP protocols. In addition, the energy consumption is higher when using AllJoyn than when using CoAP for both, transmitting and receiving devices. Nevertheless, for some receiving devices, the energy drain is very similar for both protocols. Notice that the battery consumption differs according to the device type, even when they have same role (Consumers) and protocol active. This shows the effect of the devices heterogeneity on the collaborative sensing activity.

These results can be anticipated since the CoAP protocol is very simple and it is therefore, expected to consume less energy than a more complex protocol (like AllJoyn) that includes network and service discovery functionalities. We then conducted a number of tests in order to verify the benefits of collaborative sensing. Unlike the previous tests, here we aim not only to evaluate the overall costs of collaborative sensing, but also to isolate the costs related to the data retrieval mechanisms used by the MASU framework. Thus, we intend to differentiate between the sensing costs (which are required even when the units work in a stand-alone fashion) and the costs caused by the collaborative activity itself. For these tests, we considered the worst case scenario, that is, when sharing data using the most energy-intensive protocol. Consequently, the application executing the MASU framework had only the AllJoyn protocol activated.

**Figure 16:**
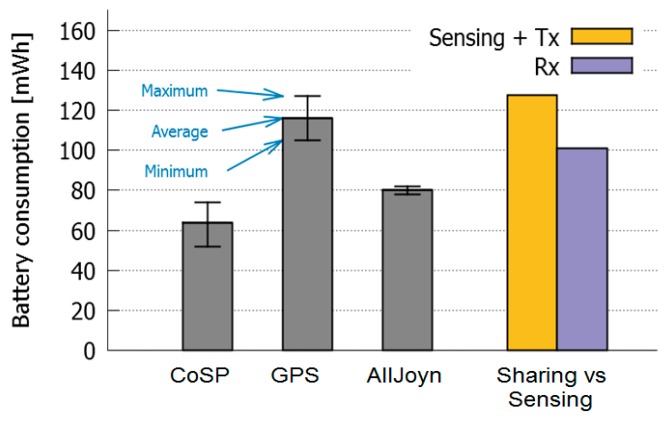
Evaluation of the cost of AllJoyn in a collaborative sensing activity.

Figure 16 shows a comparison of the maximum, minimum and average energy consumption when: (i) they only have the prototype application (CoSP) running and (ii) the devices are only sensing GPS; (iii) they have the application running and the AllJoyn service active, but they are not sharing any data. Thus, we intend to evaluate the cost of maintaining an AllJoyn session (16 mWh in average), in comparison with the cost of sensing. These results also show that the cost of sensing GPS is considerably higher than the cost of using AllJoyn.

The second set of bars in Figure 16 shows the average cost of sensing and transmitting GPS data (corresponding to a Producer role) using AllJoyn, versus the cost of receiving it (Consumer). From the figure it is clear that the cost of sensing and transmitting is slightly higher than the cost of sensing GPS, whereas the cost of receiving information is smaller (15.06 mWh in average). This result indicates that there is an obvious benefit for the receiving device (GPS Consumer), while the cost in the transmitting device (GPS Producer) is relatively low, which clearly points to the advantages of collaborative sensing in terms of social welfare because there is some benefit for the overall group of users (3.41 mWh in average). It seems reasonable to think that, if number of devices increases, these benefits could increase too. Thus, we could compensate the communication cost introduced by maintaining the AllJoyn session. Nevertheless, further tests are needed to confirm this claim.

#### 5.4.2. Battery Consumption of Producers and Consumers

Some additional tests were performed to evaluate the energy costs of Producer and Consumer roles. We compared the performance of Producers and Consumers in case of high and low intensity data sharing processes, when sharing data using AllJoyn. That is, the cost of sensing from those sensors that generate low and high amounts of data, including the costs of sharing such data at low and high data rate, respectively.

The high intensity data process was established by sensing GPS data continuously and sharing it every time geolocation value changed. This implied that the GPS data was sent continuously due to the high precision of the GPS sensor and also because the devices were constantly moving. On the other hand, the low intensity data sharing process was established by sending an audio file captured from the smartphone microphone.

**Figure 17:**
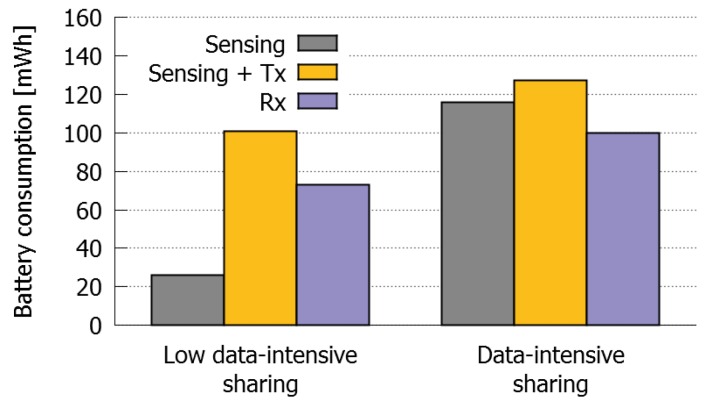
Comparison of the energy costs of high data-intensive and low data-intensive sharing processes.

Figure 17 shows the results obtained. In the case of high intensity data sharing process, the cost of sensing is slightly smaller than the cost of sensing and transmitting the data, but higher than the cost of receiving it. Therefore, in case of high data-intensive Producers we can confirm the usefulness of sharing the sensed data using AllJoyn, even when the data is shared solely with one Consumer.

Contrarily, the cost of using AllJoyn for sharing small amounts of data, at a low transmission rate, is very high and considerably higher than the cost of sensing. For this reason, in case of low data-intensive Producers it would make much more sense to collect the information directly in the devices that are going to consume it. However, it seems reasonable to think that if the number of devices is high enough, this energy cost could be compensated.

#### 5.4.3. Resource Consumption for an Increasing Number of Nodes

The evaluation of the Prototype I, provided various insights about the benefits of collaborative sensing and the involved costs. This evaluation represented a worst case scenario due to the poor efficiency of the software implementation embedded in the Unity platform, as well as due to the higher costs of AllJoyn as data retrieval method. Therefore, we conducted various tests using the prototype II, which provides a more realistic experimentation scenario with a more efficient implementation. In this case, CoAP was used as data retrieval method having only one Producer and a number of Consumers active. In this scenario, the Producer senses GPS data, creates a CoAP service and sends the GPS coordinates to the Consumers subscribed to the service.

**Figure 18:**
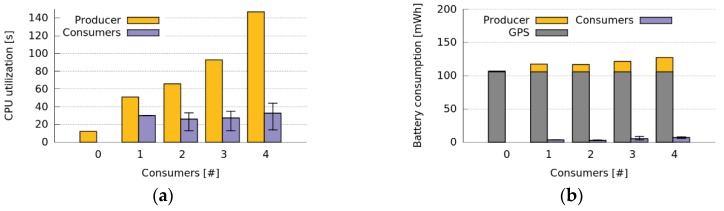
Producers and Consumers resource utilization when sharing GPS data continuously. (**a**) CPU utilization; (**b**) Battery Consumption.

Figure 18 shows the results of these tests when the Producer sends GPS sensed data to the Consumers every time that its location changes. The figure illustrates the CPU and energy consumption of both Consumers and Producers when the number of Consumers of GPS data increases. It shows that the CPU utilization of the Producer increases with the number of Consumers. This increment is mainly caused by the high number of CoAP messages that the Producer has to send due to the high change rate of the GPS data. Particularly, for each 20-min test, the Producer had to send around 900 CoAP messages to each Consumer.

We can also observe in this figure that the energy cost of the Producer (GPS sensing and transmitting) is slightly higher than the energy cost of sensing GPS, whereas the energy cost of the Consumer (receiving information) is significantly smaller. This indicates that there is a clear benefit for the Consumers, while the cost for the Producer is relatively low. Nevertheless, there is a slight battery consumption increase when increasing the number of Consumers. This was an expected result because the amount of energy should increase when more devices are added to the system, due to more the energy consumption required for the Wi-Fi subsystem maintenance. Despite this, if we consider the overall battery consumption of the system, we have an important reduction in the energy consumption (295 mWh in total for one Producer and four Consumers) when we use the prototype for sharing the sensed data, instead of sensing data independently. Once again, this shows the advantages of collaborative sensing. In this case, we achieved a 43% of reduction in battery drain, considering the overall group of devices involved. Therefore, both Consumer and Producer applications are energy-efficient and most of the energy cost of the Producer is caused by GPS sensing.

**Figure 19:**
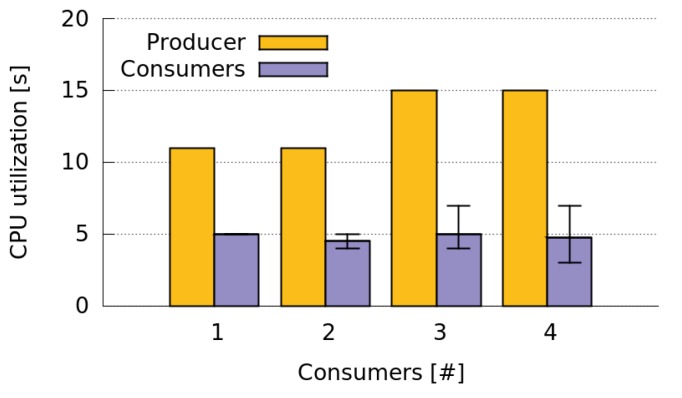
Producers and Consumers CPU utilization when sharing GPS data periodically (low data transmission rate).

We also evaluated the CPU utilization when the Consumers call the CoAP service of the GPS Producer periodically, every 60 s (Figure 19). In this case, the Producer does not send GPS data continuously. It only sends every 60 s the last GPS value measured. This means that, although the GPS sensor still active, the Producer only does a GPS reading every 60 s. Thus, the number of CoAP messages sent by the Producer decreases significantly (around 20 messages for each Consumer). As a result, the CPU utilization of the Producer is significantly smaller than when it was sending data continuously (Figure 18), and it increases very slowly with the number of Consumers.

#### 5.4.4. Resource Consumption of Storages and Relays

In order to assess the CPU consumption of the Storage and Relay roles, we performed a test where two Consumers subscribe to the GPS sensing services of a Producer and a Storage, respectively. This test is useful to evaluate the computational cost of both Storages and Relays since such a cost is only related with the rate at which Storages or Relays send data to Consumers. Therefore, it is not necessary to perform two separate tests to evaluate both types of roles.

**Figure 20:**
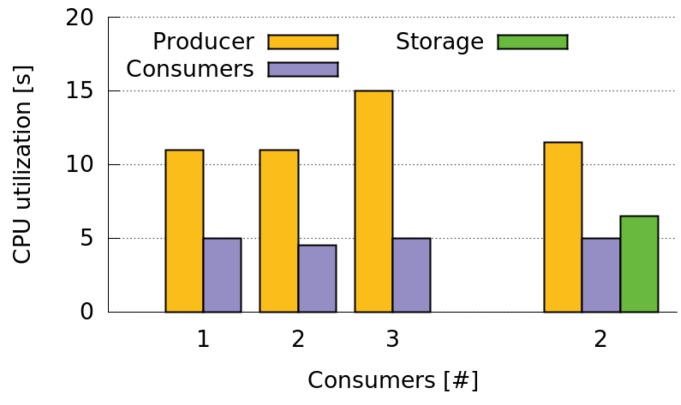
CPU utilization of Producers, Consumers and Storages when sharing GPS data periodically with two Consumers.

In this test the Storage role was assigned to the last node entering the network. This Storage receives the GPS data sensed by the Producer every 60 s and sends such data to the Consumer subscribed to it at the same rate. Results in Figure 20 show how the CPU utilization in the Storage is slightly higher than the in the Consumer but significantly lower than in the Producer. In addition, the CPU utilization of both Producers and Consumers in the case of having one Producer and two Consumers only increases slightly when we add one Storage. In this case, we have three units receiving the information generated by the Producer (i.e., two Consumers and one Relay) but at a lower cost for the Producer, reducing its CPU utilization a 23. These results indicate the usefulness of including Storages or Relays when we have two Consumers and one of them cannot receive the information from the Producer properly.

## 6. Comparing MASU with the Existing Frameworks

Although the literature reports a large number of pervasive data sensing applications and platforms that are suitable in particular application domains, there is a lack of transversal support for fully distributed and heterogeneous solutions that allow to implement pervasive sensing in variety of scenarios. Table 7 shows the similarities and differences between MASU and other sensing frameworks.

As shown in Table 7, these frameworks use some centralize component, do not support devices heterogeneity or a hybrid communication space (short and large range communication infrastructures). This is not surprising since they were proposed to support sensing in scenarios where no pervasiveness is required. In this sense, the MASU platform represents a step forward in that direction.

## 7. Conclusions and Future Work

Although the literature reports a large number of pervasive data sensing applications and platforms that are suitable in particular niches, there is a lack of transversal support for fully distributed data sensing, particularly when the sensed data need to be shared ad hoc and on demand fashion. This type of sensing can be used in various applications scenarios, like urban search-and-rescue processes, mobile learning activities and hospital work.

Trying to contribute to address such a need, this paper proposes a framework, named Mobile Autonomous Sensing Unit (MASU), which provides a set of services that allows mobile devices to perform distributed pervasive data sensing, both in an autonomous and collaborative way. The design of the framework includes the definition of different roles that can be played by a group of devices that are performing collaborative sensing activities. Developers of this type of applications can take advantage of this framework, by reusing these services and reducing thus the risks and effort involved in the development process.

The framework was evaluated using both simulations and empirical tests involving a software prototype. Both types of evaluations provided various insights on the performance of the framework under diverse hardware, networking and mobility conditions.

The simulation results demonstrated that the social nature of human interaction can benefit the performance of the framework, since people’s mobility patterns contribute to have high availability of the information sensed by the mobile devices. In addition, the simulations helped us determine the costs of the collaborative sensing, in terms of network utilization. We also showed how Mobile Ad hoc Networks (MANETs) provide an interesting communication support that provides higher network connectivity due to their multi-hop features. Consequently, we demonstrated that MANETs can also contribute to the accessibility of the information sensed. Nevertheless, the simulations also revealed that MANETs have a slightly higher cost (in terms of number of control messages), but a considerably higher cost in terms of data messages due to the unicast nature of the communication, as well as due to the higher node connectivity. These facts could derive in scalability problems of the framework when using MANETs as communication support.

Using the prototype, we evaluated the Sensing Tier of the framework. We showed the benefits of using the framework to perform collaborative sensing in terms of energy savings and social welfare in comparison to pure phone sensing, achieving a 43% energy savings for the overall system (Section 5.4.3). We also evaluated the costs associated with the use of the framework for collaborative sensing in terms of hardware resources of the participating devices. This evaluation considered some device heterogeneity since we used smartphone and tablets with diverse hardware specifications.

Results from both types of evaluation methods provided empirical evidence on the usefulness of the proposed framework. These results provided valuable insights about the benefits of the proposed framework to support pervasive sensing in terms of resource optimization as well as about its limitations and associated costs.

The obtained results in both evaluation experiences show that the services provided by the framework are suitable for supporting distributed pervasive data sensing, and also contributes to keep low the energy consumption of the mobile devices involved in the process. As a next step, we consider to develop a variety of these applications to assess more in-depth the impact of the MASU framework.

## Figures and Tables

**Table 1:** Role selection and activation mechanisms for the different role settings.

	Dynamic Centralized	Distributed
Control Tier	By leader election algorithm	All active units
Sensing Tier	By the manager	By consensus algorithm

**Table 2:** Simulation general parameters.

Parameter	Value
Simulation time	1200 s
Simulation area	360 m × 360 m
Number of nodes	40
Node model	iPhone 5
Wi-Fi standard	IEEE 802.11n
Propagation model	YansWifiChannel
Transmission power	0.66 W
Transmission range	80 m

**Table 3:** Parameters of the mobility models of the nodes.

**Random Walk**	**Value**
Maximum speed	1.5 m/s
Maximum pause	60 s
**SLAW**	**Value**
Cluster ratio	25 m
Maximum pause	60 s
**Nomadic**	**Value**
Avg. nodes per group	4
Group size deviation	1
Maximum speed	1.5 m/s
Maximum pause	60 s
Maximum distance	15 m

**Table 4:** Message setup for AP, T2T and MANET networks.

**Variable**	**AP and T2T**
Control messages	UDP broadcast
Data messages	UDP broadcast
**Variable**	**MANET**
Control messages	UDP broadcast
Unit detection messages	-
Data messages	UDP unicast
Routing protocol messages	UDP broadcast
HELLO interval	2 s
Transmission control interval	5 s

**Table 5:** Implementation details of the two prototypes.

Implementation Aspect	Prototype I	Prototype II
Implementation Type	Embedded into Unity	Native Android application
Network infrastructure	AP	T2T
Mechanism for the P2P network creation	AllJoyn	AllJoyn
Location of the Manager role	In the first node that joins	In the node that acts as AP
Data gathering method	Alljoyn	CoAP
Data message type	TCP	UDP unicast

**Table 6:** Devices used in the evaluation of the prototype.

	A1, A2, A3	B	C	D	E
Device Type	Smartphone	Smartphone	Smartphone	Tablet	Tablet
Model	HTC Desire	HTC One	Samsung Note II	Google Nexus 7	Google Nexus 7
Android Version	2.3.3	4.4.3	4.4.2	4.4.4	5.0.2
Chipset	Qualcomm QSD 8250	Qualcomm APQ 8064T	Samsung Exynos 4412 Quad	Nvidia Tegra 3	Nvidia Tegra 3
	Snapdragon S1	Snapdragon 600			
CPU	1 GHz Scorpion	Quad-core 1.7 GHz Krait 300	Quad-core 1.6 GHz Cortex-A9	Quad-core 1.2 GHz Cortex-A9	Quad-core 1.2 GHz Cortex-A9

**Table 7:** Comparison between MASU and other frameworks.

Framework/Platform	Purpose or Use	Similarities with MASU	Differences with MASU
METIS [22]	- General purpose platform. - It supports opportunistically offloading sensing by involving stationary sensors embedded in the environment. - Focused on saving energy.	- It supports continuous sensing and autonomy of the nodes. - Flexible (by gain threshold).	- It requires instrumenting the sensing area. - The only criterion used to determine the sensing strategy is energy saving.
Darwin [6]	- Collaborative reasoning framework. Uses machine-learning techniques specifically designed to run on sensor-enabled mobile phones.	- It supports device heterogeneity.	- It does not support collaborative sensing, distributed/ad hoc solutions and autonomous nodes.
CoMon [8]	- General purpose platform. - It supports cooperative ambient monitoring through opportunistic cooperation among nearby mobile users. - Focused on saving energy.	- It supports continuous and distributed sensing. - It supports nodes mobility, autonomy and sensing fault tolerance.	- It does not support device heterogeneity and one-to-one interactions. - The only criterion used to determine the sensing strategy is energy saving.
GCF [17]	- General purpose platform. - It supports opportunistic sharing of contextual information.	- It allows performing collaborative sensing involving distributed autonomous nodes. - It uses various criteria for selecting the data sensing strategy.	- Focused on grouping devices. - It does not provide device-to-device communication or support for device heterogeneity. - It does not support long-range communication infrastructures.
Remora [19]	- It implements a body sensor network. - It shares sensing information and classifiers for saving energy.	- It allows collaborative sensing involving distributed autonomous nodes.	- It does not support device heterogeneity and one-to-one or group interactions. - The only criterion used to determine the sensing strategy is energy saving. - It does not support long-range communication infrastructures.
C-SPINE [20]	- It implements a body sensor network. - It supports collaborative reasoning, computing and data fusion.	- It supports distributed and ad hoc sensing. - It supports groups of sensing devices.	- It implements collaborative sensing. - It does not support long-range communication infrastructures.
EasiSee [24]	- It implements collaborative sensing; particularly for vehicle counting and classification.	- It performs collaborative sensing.	- It supports only one-to-one interactions between stationary nodes. - It uses centralized components.
AnonySense [44]	- General-purpose platform. - It opportunistically shares anonymized contextual information.	- It supports collaborative sensing.	- It supports ad hoc sensing, but using a centralized by registry.
